# Supplementary material for: Fronto-parietal networks shape human conscious report through attention gain and reorienting
Source: Commun Biol. 2023 Jul 15;6:730. doi: 10.1038/s42003-023-05108-2 (PMC10349830; doi:10.1038/s42003-023-05108-2)
Supplement: Supplementary file 1 — Supplementary information [file 42003_2023_5108_MOESM1_ESM.pdf]

Supplementary materials for

Fronto-parietal networks shape human conscious report through attention gain and reorienting

Jianghao Liu*, Dimitri J. Bayle, Alfredo Spagna, Jacobo D. Sitt, Alexia Bourgeois, Katia Lehongre, Sara Fernandez-Vidal, Claude Adam, Virginie Lambrecq,Vincent Navarro, Tal Seidel Malkinson*, Paolo Bartolomeo*

*Corresponding authors. E-mail: jianghaolouisliu@gmail.com, tal.seidel@mail.huji.ac.il and paolo.bartolomeo@icm-institute.org

This PDF file includes:

Supplementary Figures 1 to 5
Supplementary Table 1

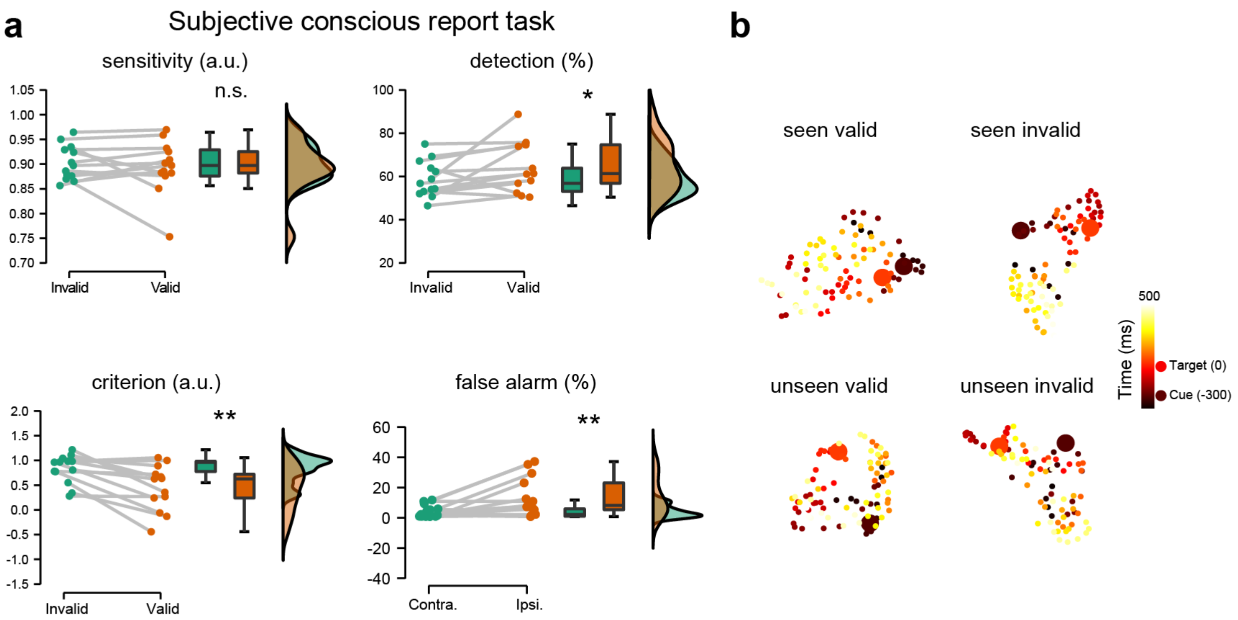

Supplementary Figure 1. Subjective report task: signal detection theory analysis and visualization of neural activity components

a. Comparison of sensitivity, detection rate, criterion, and false alarm rate in valid *versus* invalid trials for the subjective conscious report task. Dots represent individual performance. *p<0.05; **p<0.01; n.s.: not significant; a.u. arbitrary unit. Contra. (Ipsi.): (erroneously) reported targets as contralateral (ipsilateral) to the cue. False alarm rate was not computed for the discrimination task, as it is meaningless in these task settings. Boxplot shows values of median, upper quartile, lower quartile, maximum and minimum, respectively.

b. Two-dimensional t-distributed stochastic neighbor embedding (t-SNE) visualization of neural activity components of all contacts. Color map represents time points, darker for earlier and lighter for later time points.

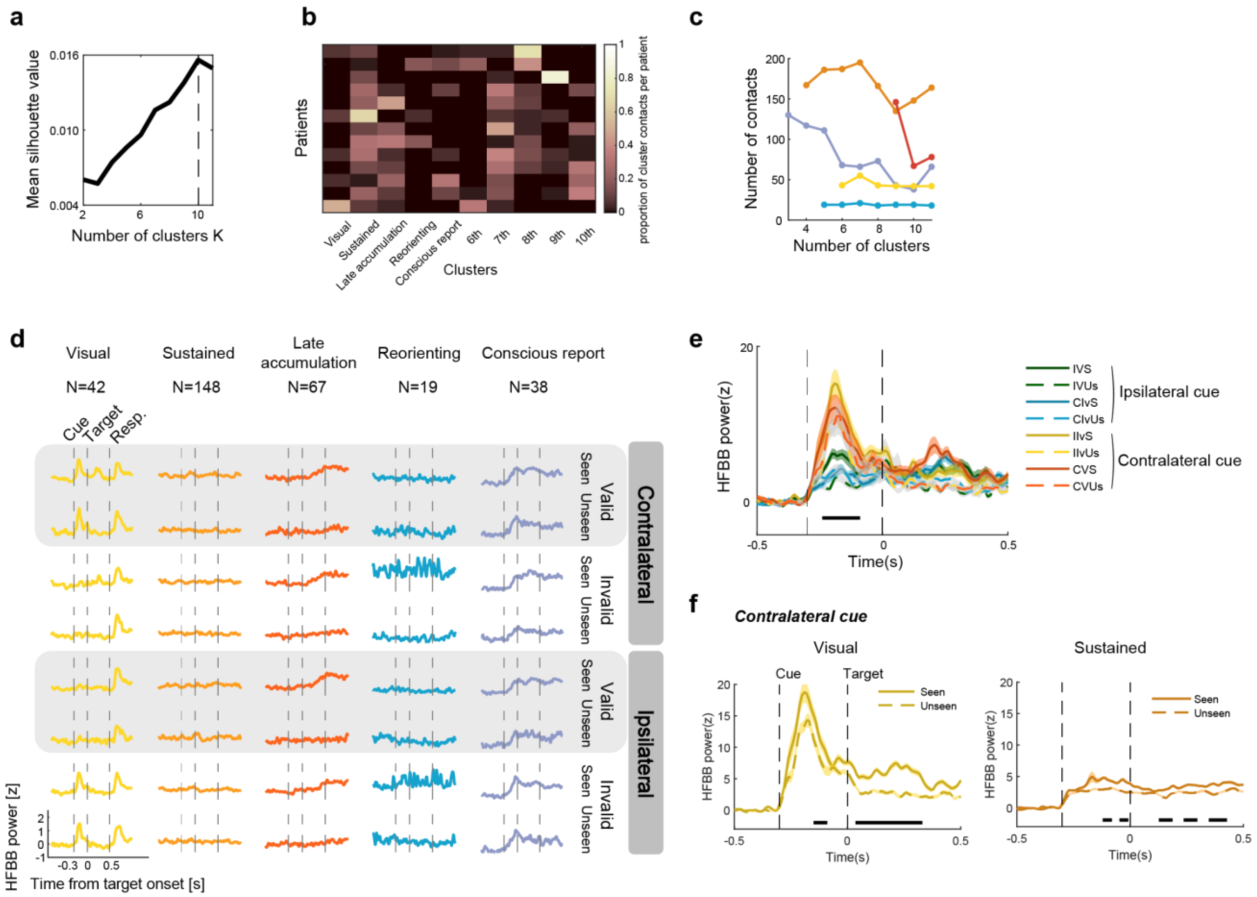

Supplementary Figure 2. Clustering the neural activities of intracerebral contacts

a. Mean silhouette value of all contacts across k-means solutions.
b. Partition of contacts by cluster for patients. Rows represent patients and color map denotes the proportion of contacts in each of the clusters per patient. This demonstrates that clusters did not result from any single participant's trajectory activity, but rather reflected temporal patterns across many participants.
c. Cluster stability. The change of the number of contacts across *k*-means solutions.
d. Visualization of trajectory neural activities by conditions for k-means solution K = 10. Resp. - response screen, after which the participants were allowed to respond. N - number of contacts in the cluster.
e. Neural activity in the Visual cluster. During the cueing period (-300ms to 0), the Visual cluster showed a target side x validity interaction, with higher neural activity for contralateral cues than ipsilateral cues. Black horizontal bar for all $p < 0.05$, Holm-Bonferroni corrected. I/C: Ipsilateral or Contralateral target; V/Iv: Validly or Invalid cued targets; S/Un: Seen or Unseen.
f. Comparison of cue-potentiated activity for conscious reporting of targets during the cueing period in the hemisphere contralateral to the cue. Black horizontal bar for all $p < 0.05$, Holm-Bonferroni corrected.

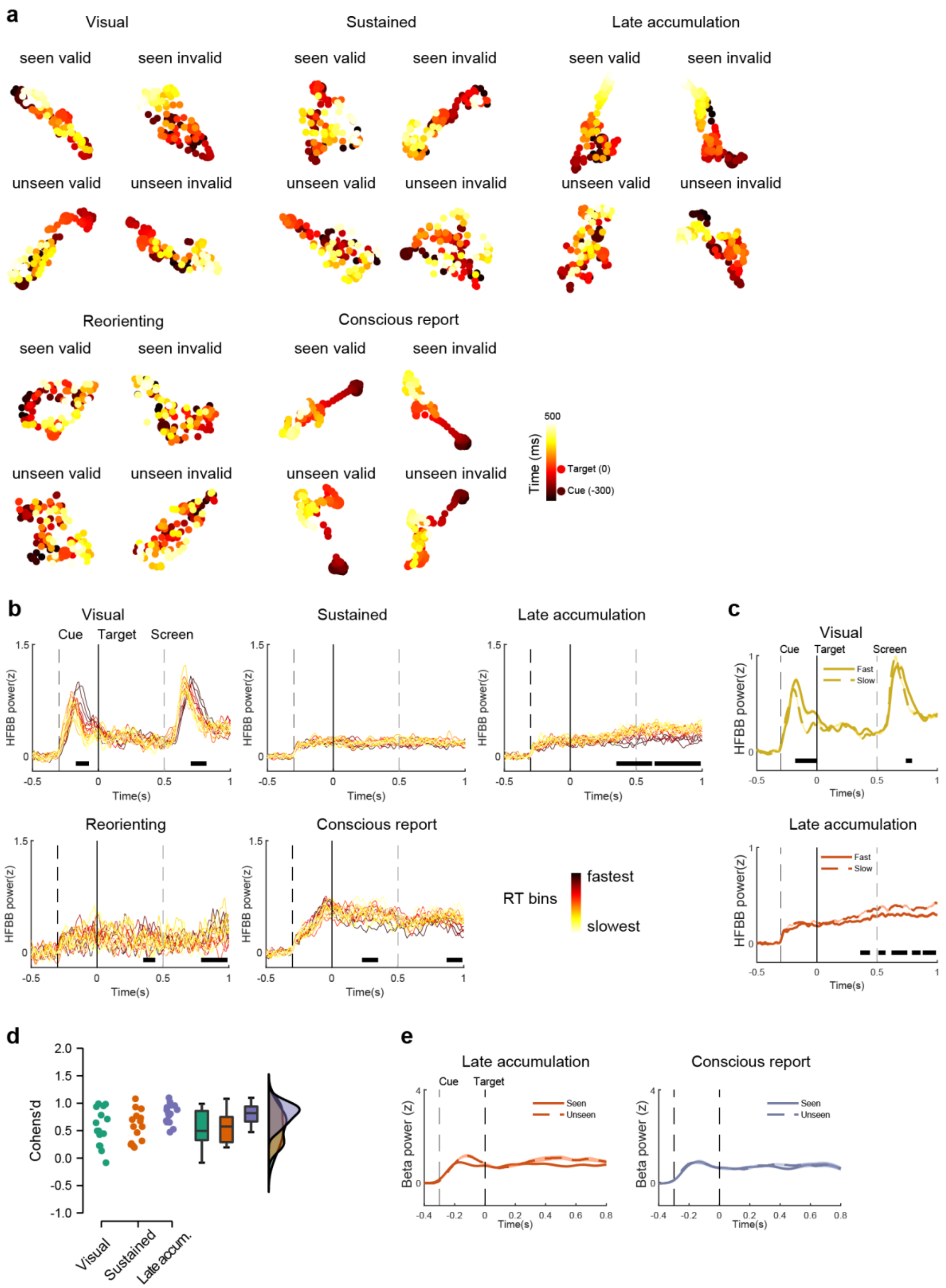

Supplementary Figure 3. Intracerebral clusters neural activity

a. Neural component visualization with t-SNE. Colormap represents time and dots represent neural activity per time point, darker for earlier and lighter for later time points.
b. Visual stimuli- and RT-modulation of target-locked neural activity in a time window from target onset to 1,000ms. In each cluster, the trials in all conditions were divided into 20 quantile RT bins of equal probability. RT bins were sorted according to their mean RT in the discrimination task from fastest (in yellow) to slowest (in red), with neural activity pooled across contacts in each cluster, and RT-related modulation was tested using a time-resolved one-way ANOVA, performed on the neural activity across RT bins. Black horizontal bars indicate $p < 0.05$, Holm-Bonferroni corrected.
c. Comparison of neural activities of the ten fastest and the ten slowest RT-bins by time-resolved *t*-test. Black horizontal bar for all $p < 0.05$, with Holm-Bonferroni correction. No significant difference was found in the Sustained, Reorienting and Conscious report clusters.
d. Linear contrast comparison of the attentional enhancement effect across the Visual, Sustained, and Late accumulation clusters. Cohen's d was derived from time-resolved *t*-test for the contrast (seen valid - unseen valid) - (seen invalid - unseen invalid) around the significant interaction time point in each cluster. Late accum. = Late accumulation cluster. Dots represent Cohen's d values at different time points. Boxplot shows values of median, upper quartile, lower quartile, maximum and minimum, respectively.
e. Beta band power. No decreased beta activity was observed in the Late accumulation and the Conscious report clusters, indicating the activity in these clusters does not reflect motor planning.

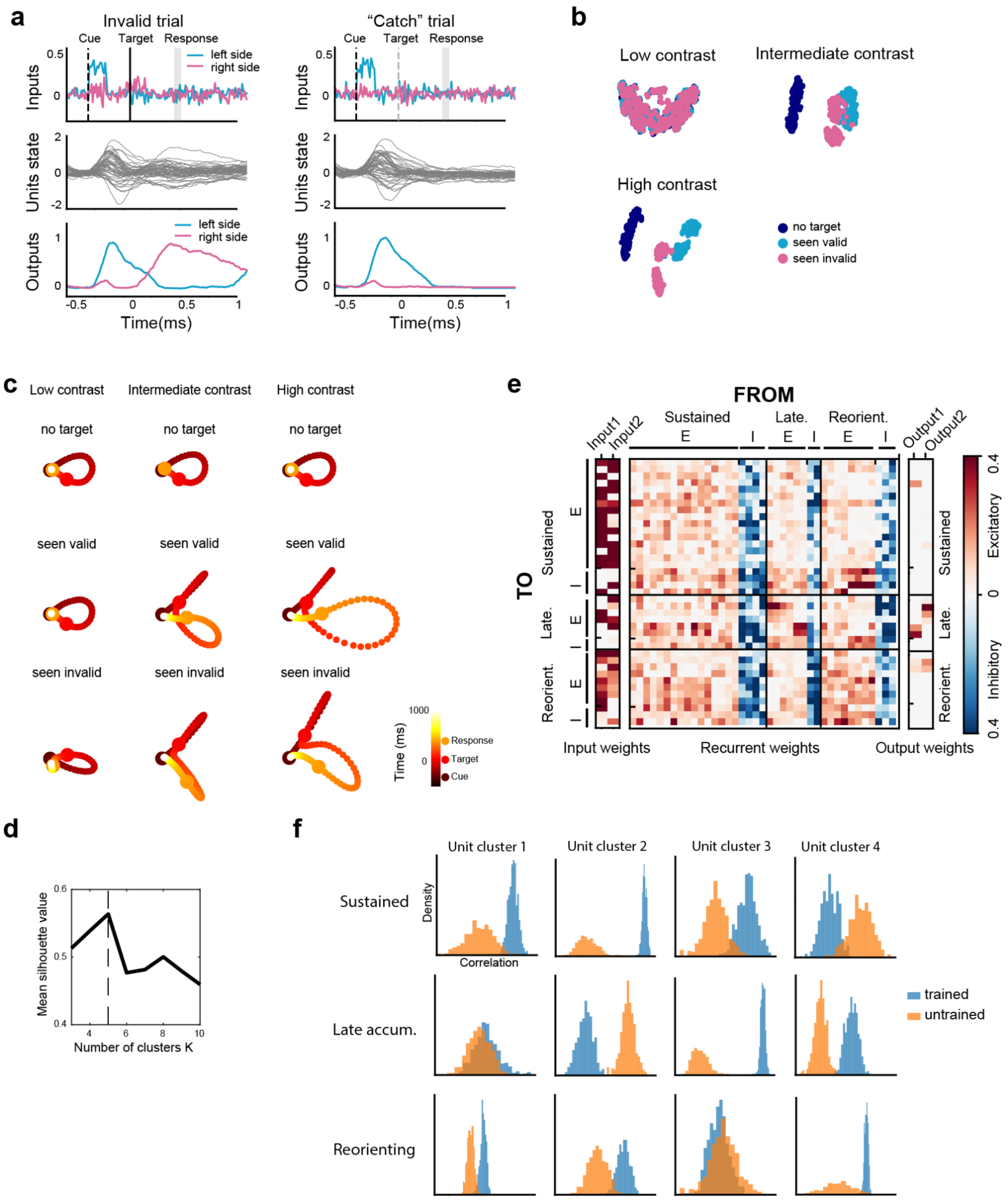

Supplementary Figure 4. Task representation and unit activity in the recurrent neural network model

a. Example of task structure in invalid (left panel) and in target-absent "catch" trials (right panel).
b. Two-dimensional t-SNE visualization of unit activity of all units, for low, intermediate and high target contrast levels, respectively. Dots represent trials.

c. Two-dimensional t-SNE visualization of unit components of all units. Colormap represents time points.
d. Mean silhouette values of all units across k-means solutions in trajectory-clustering of RNN units.
e. Directed connection weights of the task-optimized trained model. Left columns: input weights mediating sensory enhancement gain; Middle columns: directed unit-to-unit connection weights; Right columns: output weights mediating report gain). Connections go from unit columns (“pre-synaptic”) to unit rows (“post-synaptic”). Each cluster contains both excitatory (E) and inhibitory (I) units. Red = excitatory weights; blue = inhibitory weights; Late. = Late accumulation; Reorient. = Reorienting.
f. Histograms of the correlation coefficient between the trajectory of neural activity and unit activity for trained model versus untrained model. Unit cluster 1 to 4 correspond to four clusters showed significant conscious report effect. The scale was adapted to visual inspection of trained/untrained difference. Late accum. = late accumulation cluster. No significant trained/untrained difference was observed between unit clusters and the neural Visual or Conscious report cluster.

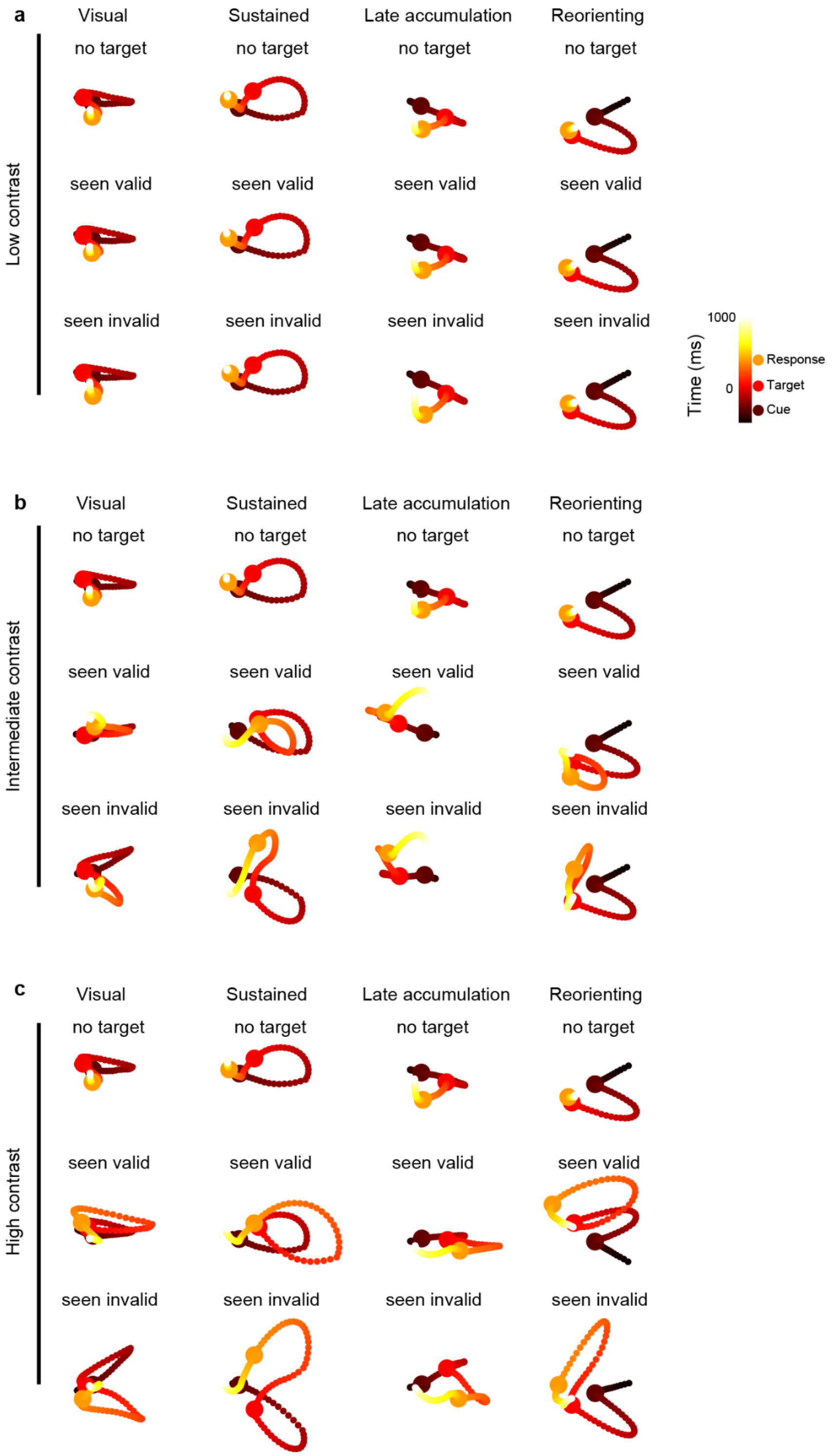

Supplementary Figure 5. Unit components of RNN clusters

Two-dimensional t-SNE visualization of unit components for (a) low, (b) intermediate, and (c) high target contrast levels by clusters. Color map represents time, darker for earlier and lighter for later time points. Difference in unit component for validly and invalidly cued seen targets emerged when target contrast attained intermediate level, and increased for higher target contrasts.

Supplementary Table 1. Localization of the implanted contacts according to the Desikan-Killiany-Tourville atlas.

Region name	Implanted Electrode N	Left	Right	Visual N	Sustained N	Late accumulation N	Reorienting N	Consciousness N
Banks superior temporal sulcus	4	1	3	0	1	0	0	0
Caudal anterior-cingulate cortex	11	3	8	0	2	0	0	0
Caudal middle frontal gyrus	20	2	18	0	2	3	0	2
Cuneus cortex	1	0	1	0	0	0	0	0
Entorhinal cortex	5	3	2	0	1	0	0	0
Fusiform gyrus Posterior	21	7	14	4	2	0	0	6
Fusiform gyrus Middle	16	7	9	2	3	1	0	2
Fusiform gyrus Anterior	4	3	1	0	3	0	0	0
Inferior parietal cortex	39	3	36	9	8	1	6	4
Inferior temporal gyrus Posterior	16	6	10	2	4	2	0	0
Inferior temporal gyrus Middle	17	3	14	0	5	1	1	0
Inferior temporal gyrus Anterior	22	9	13	0	10	0	0	0
Lateral occipital cortex	12	0	12	0	1	0	1	0
Lingual gyrus	15	0	15	0	2	0	0	0
Medial orbital frontal cortex	12	5	7	0	2	4	0	0
Middle temporal gyrus Posterior	61	7	54	13	15	4	3	2
Middle temporal gyrus Middle	30	10	20	0	13	1	1	0
Middle temporal gyrus Anterior	30	20	10	0	2	0	0	0
Parahippocampal gyrus	7	3	4	0	3	3	0	0
Paracentral lobule	5	2	3	0	1	0	0	0
Pars opercularis	10	5	5	0	0	1	0	2
Pars orbitalis	31	12	19	1	3	6	2	2
Pars triangularis	8	0	8	0	1	3	1	0
Pericalcarine cortex	1	0	1	0	0	0	0	0
Postcentral gyrus dorsal	5	5	0	0	0	0	0	0
Posterior-cingulate cortex	6	6	0	2	0	1	0	0
Precentral gyrus dorsal	15	12	3	0	0	3	0	6
Rostral anterior cingulate cortex	4	0	4	0	1	0	0	0
Rostral middle frontal gyrus	13	7	6	0	1	3	0	0
Superior frontal gyrus	73	12	61	0	11	18	0	5
Superior temporal gyrus Posterior	33	17	16	1	7	0	3	2
Superior temporal gyrus Middle	22	19	3	0	4	0	0	0
Superior temporal gyrus Anterior	25	20	5	0	6	0	0	0
Supramarginal gyrus	22	7	15	0	1	4	1	3
Temporal pole	19	11	8	0	10	0	0	0

Postcentral gyrus ventral	6	6	0	0	1	0	0	0
White matter	62	38	24	8	14	5	0	2
Hippocampus	24	16	8	0	8	3	0	0
**Total**	**727**	**287**	**440**	**42**	**148**	**67**	**19**	**38**
**Frontal**	202	60	142	1	24	42	3	18
**Parietal**	83	29	54	11	10	5	7	6
